# Genesis of the Heroin-Induced Addictive Process: Articulation Between Psychodynamic and Neurobiological Theories

**DOI:** 10.3389/fpsyt.2020.524764

**Published:** 2020-12-09

**Authors:** Hélène Scarna

**Affiliations:** ^1^Centre de Recherche en Psychopathologie et Psychologie Clinique, Université Lumière Lyon 2, Bron, France; ^2^Laboratoire de Psychologie EA 3188, Université de Bourgogne Franche-Comté, Besançon, France; ^3^Univ Lyon, Université Claude Bernard Lyon 1, Inserm, Stem Cell and Brain Research Institute U1208, Bron, France; ^4^Centre de Soin, d'Accompagnement et de Prévention en Addictologie, Hôpital de la Croix-Rousse, Lyon, France

**Keywords:** addiction, narcissistic vulnerability, true self/false self, psychodynamic theories, neurobiology

## Abstract

Psychotherapeutic consultations of drug addict's patients in a Care, Support and Prevention Center in Addictology led us to propose several hypotheses on the genesis of addiction and its articulation with currently available neurobiological data. This care center dispenses both pharmacological maintenance medications for heroin dependence, such as methadone or buprenorphine, and psychological support. Our first hypothesis posits that the addictive process is driven by the narcissistic vulnerability of these patients, its neurobiological foundations being mainly mediated by the activation of endogenous opioid systems. Drug use/abuse could be a way to make arise the “True Self,” therefore overcoming the defensive system's set up to protect oneself from early traumas. The neurobiological impact of traumas is also developed and articulated with psychodynamic concepts, particularly those of Winnicott. Additionally, functions of addiction such as defensive, anti-depressant roles and emotional regulation are discussed in relationship with their currently known neuroscientific bases. Although the experience in the psychodynamic clinic is at a level of complexity much higher than what is currently accessible to the neurosciences, most of the research in this domain stays in line with our psychological understanding of the addictive process. Finally, we outline some critically sensitive points regarding the therapeutic support.

## Introduction

Several hypotheses relating to the phenomenon of addiction have been proposed. Some authors have developed the incentive sensitization theory which corresponds to the drug-induced sensitization of brain mesocorticolimbic dopaminergic systems attributing incentive salience to reward-associated stimuli (1). This can induce a pathological incentive motivation for drugs. Thus, the will of drug addicts is taken over by chemicals (2). Nevertheless, other authors have suggested that the temporally myopic drug user makes voluntary self-destructive choices as a function of contextual variables. They recognize that brain changes underlie the development of addiction but that the controlling variables for addiction are not solely biological (3). One assumes this behavior as a compulsive illness difficult to counter, others as involving a desire-motivated choice, i.e., something that takes place within the domain of ordinary intentional action (4). However, these two views are paradoxically not incompatible and the addiction may also come from a vulnerable background as outlined in this article.

Our clinical experience with patients attending a Care, Support, and Prevention Center in Addictology (CSPCA) allows to examine the addictive problematic and to articulate it with the corresponding biological knowledge, thanks to our dual practice of clinical psychologist and neurobiologist. More specifically, we will apprehend this issue from both psychodynamic and neurosciences perspectives.

Clinical observations of drug-addicted patients reveal a significant frequency of narcissistic suffering following early wounds of self-esteem, emphasizing that narcissistic vulnerability is one of the roots of the addictive process. Accordingly, it is in the historical constitution of narcissism, or its double, the self, that it is undoubtedly necessary to look for one of the causes of the recourse to this behavioral solution taking in place to protect oneself from the psychic traumas previously undergone.

The paradox of addictive behavior raises numerous questions. The pleasure is put forward during the consumption of the first doses of the drug; but then the acute rewarding effects decrease and yet dependent people will continue to use their favorite product despite a growing number of negative consequences. Of course, the withdrawal syndrome comes into play, with both physical and psychological pain. Although maintenance treatment, methadone and buprenorphine, are put on place to prevent this, some patients divert the medical use of these therapies by inhaling or injecting them. They often explain that this behavior is necessary to them, while putting their life in danger. Thus, the question arises of the psychic function of addiction to better understand this pathology and improve its management/treatment.

## The Population Studied

Two hundred and thirty eight patients (91 women and 147 men), aged from 16 to 75 with an average age of 31 years, received psychological supports. Most were poly-addicts, but considering the product consumed primarily: 73% were heroin addicts, 6% smoked cannabis, 5.5% were cocaine users, 4% had a problem with alcohol, 3% were addicted to psychotropic drugs and 0.5% were consuming ecstasy. As for the remaining 8%, they had addiction without product (gambling, shopping or sex). The older ones were concerned with gambling addiction or compulsive shopping. The interviews are conducted face-to-face (sometimes side-by-side) with a variable frequency. We have encountered some patients only once, others for more or less long follow-ups, some lasting more than 10 years but including interruptions.

## Definition of Narcissism

World Health Organization (WHO) defines health as “a state of complete physical, mental and social well-being”; mental well-being is linked with biological factors and must be understood as arising from the development of the individual since conception. At the psychological level, one may assume that well-being has its roots in primary narcissism, in the spontaneous gesture of the baby (5). We need therefore to understand the emergence of narcissism.

### Prenatal Phase

Grunberger (6) hypothesized the existence of a primitive narcissistic nucleus that would be the trace of fetal cenesthesia. It represents an organic experience that would leave its mark on any individual during his whole life.

At the somatic level, fetal sensoriality develops during the first weeks of life. The emergence of exteroceptive and proprioceptive sensorialities follows a chronological order: first touch (7), then olfaction and taste, proprioception, hearing and finally vision. Sensory systems are functional before birth as newborn recognizes, for example, the sounds heard *in utero* and especially his mother's voice, even in the premature baby born at 6 months of pregnancy (8).

To explain how the prenatal cenesthesic state shows, after birth, a positive valence (meaning to be a source of well-being), it is necessary to formulate a complementary hypothesis, namely that the brain of the fetus is also armed to associate with this state an attractive value and not only neutral. In the adult, the hedonic impact of a stimulus involves complex neurobiological events that are difficult to disentangle from those underlying the motor and cognitive implications of this affect, particularly with human brain imaging. However, high-precision neuroanatomical studies in rodents have reported that the activation of mu-opioid receptors in inter-connected subcortical structures of the limbic system is mediating non-conscious pleasant coloring of a sensation (9). These regions include the nucleus accumbens and ventral pallidum. Thus, the endogenous opioid system seems to be crucial in the attribution of a pleasant valence to a sensation. Panksepp's group showed that attachment processes (10) in the young puppy or juvenile guinea pig were underpinned by endogenous opiates and were thus a source of well-being (11). In addition, a study in mutant mouse pups with a deletion of the gene expressing the μ-opioid receptors showed a deficit in their attachment behavior to their mothers, while the latter, themselves, behaving normally with their child. Separated from their mothers, genetically modified mouse pups emit much less distress calls than normal mouse pups, while they emit more when confronted with cold for example. In the presence of several mothers, they do not have a preference for their progenitors. It appears that μ-receptors play a key role in the attractiveness of maternal stimuli (12). Indeed, the attribution to the prenatal experience of a positive valence seems necessary for the establishment of the child's attachment to his mother.

In humans, opioid systems appear as early as the 19th week of life after conception (13), which makes it plausible for their involvement in assigning a positive valence to prenatal sensoriality. Of note, “emotional” touch data seems to support this possibility (7). The sensoriality of the skin is indeed multiple: besides its discriminative function allowing us to manipulate the objects and to detect their tactile properties and their temperature, it has an affective function as illustrated by the pain when the skin is damaged or conversely by pleasant sensations when the touch is soft. This somatosensory information is conveyed to the brain by different types of fiber. Those responding to touching/caressing project to the limbic cortex (ventromedial prefrontal cortex and posterior insula) and the nucleus tractus solitarius (NTS), and induce an emotional response (14). Furthermore, in human, this social touch is show to activate the μ-opioid system of ventral striatum, anterior cingulate and insular cortices (15). Additionally, NTS is known to be directly linked to the paraventricular nucleus in the hypothalamus and its activation can release oxytocin, and interestingly, several studies highlight the involvement of oxytocin in the empathy and socio-cognitive processes (16).

From a developmental point of view, Bystrova (17) proposes that fetal movements in the amniotic fluid produce oscillations of lanugo (fluff covering the fetus' body) likely to stimulate the light touch-sensitive fibers, whose function would be to activate brain regions such as the insular cortex. This prenatal “massage” would have a pleasant and soothing effect, mediated probably via opioidergic and oxytocinergic systems, and promoting fetal growth.

### Postnatal Phase and Early Parent-Infant Relationship

The repetition of the experience of narcissistic completeness during the postnatal period (i.e., when the infant receives sensations close to those already experienced antenatally) can therefore be thought of, not only as a reinforcement of the prenatal cenesthesic trace, but also as an enrichment of the possibilities of activation of this trace by other channels through postnatal sensorimotor contributions.

Winnicott (18) has used very few times the term “narcissism” but he insists on the fact that it is easy to relate to, such as the integration of the elements of the personality to make a whole, leading to the constitution of a self. Moreover, Grunberger (6) and Winnicott (19) emphasize the role of the environment in the construction of child narcissism, the primary object whose first function after birth is to ensure the continuity or rather the repetition of the experience of fetal narcissistic completeness. Importantly, two notions proposed by Winnicott for defining early maternal care are the “holding” and the “handling.” The “holding” is both a physical and an emotional act (20). It is expressed through the way the mother carries, feeds, speaks and responds to her baby and in her understanding of his needs and experiences. The mother's “handling” is describing as her sensitive touch and responsive care of the baby's body which will enable him to experience physical and emotional satisfaction in an integrated way. This helps the baby to bring together the worlds of sensation and emotion and build a stable unity of mind and body. The individual who has received enough sensitive handling in early life will experience his mental, emotional, and physical capacities as connected, personal, and authentic to the self (21).

Thus, close supportive relationships are known to enhance well-being and strengthen narcissism but what about the neurobiological bases of this state of facts? Oxytocin has an important role in this mediation and even presents a major function at the beginning of the life because this neuropeptide promotes both the preferential attention and the respective attractiveness between mother and baby and therefore the establishment of the processes of secure attachment (22). The release of oxytocin is caused, in both partners of the relationship, by breast-feeding, but also skin-to-skin contact, then all close and warm interactions between humans (23). This neuromodulator promotes the establishment of the mother-child bond, and then all other lasting social relationships, via its action on the nucleus accumbens (24). Attachment has a natural rewarding property and oxytocin increases endogenous opioid transmission (25, 26).

One may assume that an articulation can therefore be proposed with psychodynamic thinking: the benefit to the child of winnicottian ‘holding’ and ‘handling’ could involve the oxytocinergic and opioidergic systems at the biological level.

In rodents, it has been shown that one of the functions of oxytocin in the brain is to sensitize the auditory cortex of mothers to the distress calls of their pups when they stray from the nest. By finely modulating cortical plasticity, oxytocin therefore increases the perceptive salience of social acoustic stimuli of the mother, which promotes her behavior of recovery from young mouse to bring him back to his nest (27). Such behaviors are reminding of Winnicott's concept of “Primary Maternal Preoccupation” (19), defined as the identification of the mother with her infant, allowing her to know what the infant is feeling and so being able to provide almost exactly what the infant needs in the way of holding and in the provision of an environment generally.

## Early Psychic Traumatisms

Childhood trauma can have different origins: they can be confronted with neglect, abuse, but also early relationships that are more subtly maladaptive. I will focus here particularly on the latter often passing unnoticed and leaving no trace in the explicit memory.

### Early Traumatisms as the Origin of Pathological Defenses

Winnicott (20) considers that beyond the occurrence of a traumatic event circumscribed in time, it is the inadequate mode of ordinary response of the environment to the infant's ego-needs, in the long run, that may be traumatic. When the mother's adaptation is not “good enough” (28), there may be impingement and the infant secretes a “false self” that submits to the demands of the environment and develops artificial relationships (5). In appearance, this false self can be perfectly adapted to the external reality, but the psyche then dissociated from bodily experience. The false self is a defense mechanism that hides the true and protects the subject from annihilation, but creates a sense of unreality or inanity. Ultimately, these narcissistic traumatisms have the potential to cause complete paralysis of all spontaneity and all work of thought, but the infant must react to the demands of the environment. Winnicott (29) writes: “Reacting at this stage of human development means a temporary loss of identity. This gives an extreme sense of insecurity...” The reactions of the individual to environmental impingement are at the origin of the most primitive defense mechanisms such as splitting or projection. Other potential traumatic situations are described by this author, this is the case of an unpredictable environment, but also what could have been useful and did not happen in the first moments of the individual (30). The emptiness feeling that results contributes to the development of a flaw in the organization of the ego. These traumatisms of the non-happened, as Roussillon (31) calls them, are traumatisms due to lack of investment, care, attention, but also of reflexivity from those who around the individual. Thus, “if the object, the mother, is too unpredictable, unavailable, elusive, unattainable, insensitive, impassive, inanimate, etc., she is out of tune, disjointed both bodily and emotionally. The baby cannot 'create' her, he cannot build the illusion that he is at the origin of his own satisfaction, he cannot share his emotional experience, cannot constitute his mother as a 'mirror' of his feeling of being. More exactly, he has the feeling of being at the origin of a 'bad' world, bad because unintelligible, discordant: he feels 'bad', in both senses of the word, namely that is to say that he feels 'bad' in what happens, which remains strange, and that he feels awful, at the origin of the evil, at the origin of the dissatisfaction which he experiences and of the loss of the harmony of the world. This is probably the root of primary guilt, the one that precedes any ambivalence” (32). The impairment is narcissistic, it is not frustration, but rather a loss of the ability to be or feel be. These subtle interpersonal traumatisms make narcissism vulnerable and open the door to solutions that can strengthen this vulnerability.

### Biological Impact of Early Traumatism

In the field of biology, the traumatism was defined from the concept of stress (33). Stress is the state of alertness and tension aroused by event of endogenous or exogenous origin and generating excitement. Biological and behavioral responses are triggered for a return to homeostatic balance of the internal medium. The arousal regulation encompasses two types of control. The first involves the autonomic nervous system and the adrenal medulla with the secretion of adrenaline, it is short-lived. The second, more sustained, engages the adrenal cortex with the release of cortisol under the control of the hypothalamic-pituitary-adrenocortical (HPA) system. In its meaning of traumatism, the definition of stress must therefore be restricted to stimuli that exceed the body's regulatory capacities (34). It must be pointed out here that the younger is the individual confronted with stress, the less he will be able to anticipate and control it by suitable physical or psychological action, which will increase his sensitivity. However, biological responses to stress can be alleviated by the presence of a relative, called the social buffering of stress. Attachment is the key factor in social buffering. Thus, maternal care, when appropriate, regulates emotional reaction to stress of children (35). This involves the release of oxytocin by the hypothalamus generated by somatosensory stimuli, like touch contact (23), in the direction of several structures responsible for the stress response such as amygdala, raphe and locus coeruleus. A second type of process, neurocognitive ones, could be involved in the emotional regulation by a third party. It is the activation of cerebral regions that respond to safety indices, such as the ventromedial prefrontal and posterior cingulate cortex (36), which in turn inhibit the fear circuit including the amygdala (37). However, premature absence of the buffering function of primary objects has a long-term biological influence. The most sensitive periods correspond to the moment of rapid maturation of the brain, a period of great plasticity facilitating the influence of the environment. These are time windows during which specific experiences are necessary for the normal deployment of a particular brain function i.e., windows of opportunity allowing the beneficial action of enriching experiences, but at the same time, there are windows of vulnerability concerning the negative effects of deleterious experiments. Each region of the nervous system has a particular developmental trajectory, so that their periods of vulnerability occur at different ages (38).

Studies in both humans and animals show that the impact of early traumatisms on the brain and its regulatory outputs (including the neuro-vegetative, endocrine and immune systems) persist into adulthood (39). These biological consequences translate into a greater sensitivity to stress and an increased risk of developing psychic and somatic diseases. Thus, the idea that a vulnerability of the adult to various pathologies (ranging from cardiovascular or metabolic diseases to anxio-depressive or psychotic states) could be biologically “programmed” or “printed” during the fetal period was proposed in 1995 by Barker (40) and is currently widely accepted by biologists. Notably, it was later extended to the early postnatal period and gave rise to the concept of “perinatal programming,” which accounts for the influence of environmental factors in the development of biological systems (41). Its mechanisms are epigenetic in nature, but also often depend on the pre-existence of a particular polymorphism of certain genes. For example, in rats, subtle stress occurs when the level of maternal care (licking/grooming/feeding) is low. Fewer the contacts with the mother during the first postnatal week are, higher is the behavioral and neuroendocrine responsiveness to stress of the offspring. Cross adoption reverses maternal effects on the offspring, showing that there is a direct relationship between the quality of maternal care and the phenotypic development of the offspring (42). Moreover, a study in children raised in orphanages then adopted shows an increase in the volume of their amygdala compared to those of children raised in their families (43). In less severe cases of maternal deprivation, such as depressed mothers, children also showed increased amygdala and high levels of glucocorticoids (44). Amygdala of these children is solicited prematurely for associative learning of negative stimuli with a motivational relevance, which disrupts their development. Indeed, the intracellular signals promoting the neuronal plasticity intervene not only during the neuronal development but also during the learning. Thus, trauma accelerates the closure of the sensitive period of plasticity in the amygdala, which may decrease connectivity, occurring later, and with cortical afferents inhibiting its responsiveness (45). This type of change may support what Winnicott argues about the traumatic nature of what was lacking in the individual's primitive experiences.

## Primary Hypothesis

The hypothesis of a first link between the neurobiological and psychologic aspects involved in the appearance of a drug addiction is as follows: knowing that heroin activate endogenous opioid systems, this exogenous product would actualize the cenesthesic traces formed during prenatal life and thus chemically replicate the state of narcissistic completeness experienced by the subject before the birth. Indeed, the activation of this peptidergic system allows the subject, at least during the first consumptions, to overcome vital needs, relieve all suffering through the analgesic function of opioids and find again, a state of well-being close to that already lived *in utero*. It is, for example, the emotional fullness of the heroin with the impression of being distanced from the environment, protected from all excess of excitement and of the psychic impingement by the entourage. From the outside, this gives the impression of perfect indifference to the problems of the surrounding world. Based on this postulate, I would put forward a hypothesis involving postnatal life to account for the addictive risk among narcissistically vulnerable personalities. I propose that the salience, and therefore the psychic impact, of the first coenesthetic experience with drugs would be all the more marked as the postnatal experiences of narcissistic completeness were rare and/or not subject to the work of symbolization. If the initial prenatal experience could not be repeated after birth so that its perceptive trace can be transformed by the mediating presence of another one, the drug will re-actualize this raw trace and will give an impression of revelation to the drug experimenter. This experience with the product would then be likely to be repeated, eventually evolving into a compulsive behavior.

Conversely, the variety of integration experiences of the self during the postnatal period and the quality of early interactions that promote primary symbolization will provide protection against the addictive risk. Indeed, in this first developmental stage, not only does each experience of satisfaction re-actualize the state of completeness, but also directs, in the subsequent stages, the drive of the child on the side of the fulfillment by himself of these experiences of pleasure (autoeroticism), a process that involves epistemophilic drives and leads from dependency to independence. Moreover, some authors, proposing a reflection on the experience of pleasure, point out that the self-related processing induces neural activity in the same brain regions that are recruited by various rewards (46). Thus, both reward processes and self-relatedness might share a similar evaluative process.

The corollary hypothesis thus considers that the failure of the construction of secondary narcissism, which prevents the subject from extricating oneself sufficiently from primitive dependence, will promote future dependence on drugs. Indeed, during adolescence, the encounter of the latter will come at the right time to make up for the deficiency of parental objects and to give the subject the illusion of empowerment.

Finally, the concepts of true- and false-self (5) provide one-step further in the understanding of the disinhibiting function of drugs, a function corresponding to a lifting of the defenses constituted by the false self. Indeed, at the beginning of life, the experience of narcissistic completeness allows the emergence of the spontaneous gesture from the True Self, whose destiny will depend on the way it is greeted by the environment. Thus, if the True Self cannot develop because of an inadequate adaptation of the environment to the infant's drives, it will be masked by the secretion of a false self. In this context, I would argue that for the consumer, drugs are a way to get in touch with his True Self and, if he has not found any other way to achieve this, the repetition of this experience would become compulsive so to keep alive the feeling of existence. This function of maintaining the contact with the true self seems to be in the foreground in patients confronted with anxiety-related feelings of internal emptiness and inanity of their existence. On the other hand, if this modality of reunion with the True Self makes it possible to maintain the feeling of existence, the addiction, which follows from it, leads at the same time to the exclusion of the object. The addiction thus reveals the failure of the encounter with the object and, consequently, the establishment of the autarkic functioning that follows.

The clinical observation shows that drug users, as long as they have not developed a dependence, often report having recreational use, only in search of pleasant sensations. Thereafter, when they become addicts and although the withdrawal symptoms and the procession of deleterious effects of the dependence set in, their words remain ambivalent, acknowledging the harmful nature of their behavior, but declaring that they cannot do without drug. This attitude is not consistent with the pleasure principle, even if its primacy was convened by the drug user to justify the initiation of his/her consumption.

At the manifest level, the destructive effect of some addictive practices, such as injection, seems to be in the foreground. However, the patients we met quickly convinced us of the irrelevance of the hypothetical death drive, and its self-destructive effects, to shed light on the addictive problematic, at least for those who resort to substitution treatment.

The initial hypothesis comes from a clinical observation from a patient chronically addicted who was followed for several years and under maintenance medication. Listening to this patient, nostalgic for heroin use, allows us to re-examine the traditional view of addiction. He talks about his morning sniff of heroin, which allowed him to get up, go to work and endure repetitive tasks that his position entailed and interact with others with ease and good humor. He therefore puts forward, not the orgasmic side of the pleasure claimed in the first place, but rather the “normalizing” effect of the well-being procured by the drug. When asked if he feels that he is the most himself, with or without heroin, he answers that the heroin allows him to be really himself, to express what he really is This initial observation led us to propose the hypothesis that one of the functions of drug use is to lift the rigid defense mechanisms that constrain the self.

## Neurobiology of Addiction

Addictive disorders are etiologically complex conditions that result from multiple genetic and environmental risk factors. Genes involved in vulnerability to addictions include both substance-specific genes and genes that act on common pathways involved in addiction to different agents and propensity to other psychiatric disorders. Substance-specific genes, including genes for drug receptors such as μ-opioid receptor, as well as genes influencing diverse aspects of addiction neurobiology (anxiety, impulsivity, reward…), have been implicated in the shared genetic liability between addictions and other psychiatric diseases. Genetic influences modulating the risk of substance use disorders vary across lifespan (47).

In individuals who are vulnerable to addiction, repetitive exposure to the agent induces long-lasting neuroadaptative changes underlying tolerance, craving, withdrawal, and leading to a motivational shift. Thus, drug addiction represents a dramatic dysregulation of motivational circuits involving changes in dopamine and opioid peptides in the basal ganglia, and in turn, the loss of control by glutamatergic afferent projections from the prefrontal cortex and insula, and finally, the recruitment of brain stress neurotransmitters, such as corticotropin-releasing factor and dynorphin, in the extended amygdala (48).

Nevertheless, the clinic leads us to ask the question of the functions of addiction. What is the point of using drugs?

## Functions of the Addiction

The search for pleasure is the function that appears first concerning drug use. Taking drugs would be a way of finding pleasure without going through the object and without fear of his reprisal. There is then disinvestment of the object.

However, this pursuit of pleasure masks many other (sometimes unconscious) goals in drug use, such as a defensive function. I have previously emphasized the disinhibiting effect of drugs that come to lift defenses built in the form of false self. This type of defense correspond to a submission to the impinging object. Such a submission does not allow the True Self to express itself. In this case, the drug not only allows finding a connection with the True Self, but also serves to recreate a protective envelope against external excitations. In this way, the subject would regain control of his inner world, somehow by appropriating the new defensive system he sets up.

On the other hand, there may have been violent rebellion against the object in the form of an uncontrollable emotional outburst, and in this case, drug use would seek to protect the ego from its passions, which constitute traumatic internal excitations. This is the hypothesis of an affective regulation function. To appease one's internal violence or to animate what in itself gives one the feeling of existing, in both cases through drug use, provides the impression of a control of one's behavior.

A last function may be considered, namely the anti-depressive effects of drugs of abuse. Depression is a malaise against which addicted patients struggle for fear of falling into an unsustainable narcissistic breakdown. Taking drugs is the last solution found to escape it, until the constraints of addiction are such that they relapse into secondary pathological depression that leads patients to seek alternative treatment.

### Affective Regulation

According to their story, addicts have to face two types of emotional state: Those who have experienced massive and/or late traumas have difficulty controlling their violence (directed against others or against themselves) and those who have undergone early and/or subtler traumas have managed to put up defenses that cut them off from their experiential lives and impoverish their fantasy world. It is therefore, on the one hand, a state of excitement linked to traumas that would still continue today (especially in a context of precariousness) and, on the other hand, a state of internal emptiness resulting of an early suppression having driven away all emotion. Parat (49) describes the mechanism of suppression as “a work of the ego, to avoid suffering and displeasure, which effects a disarticulation between affect and presentation, the goal being to get presentation to become neutral.” The sentiment of permanent insecurity (“Abandonism”) in subjects suffering from narcissistic pathologies seems to be related to the feeling of agonizing emptiness and the absence of wish.

In the first case, psychotropic drugs would be a surrogate for protective shield, the construction of which would have failed during the early interactions between the child and his relational environment. Khantzian (50) suggests that “rather than simply relieving suffering that is unsustainable, persons who abuse substances often use drugs to control their feelings, especially when they are nameless, confusing, and beyond their control.” In the second case, drug use would fill a feeling of emptiness, due to the absence of phantasmal life and source of fundamental boredom, which would make it possible to feel alive. Corcos et al. (51) use the concept of alexithymia which would constitute a defense mechanism of protective shield of affects and presentations. Drug use is therefore discovered as a way to appease violence itself or to awaken feelings capable of living a more authentic life, leading delusively to freedom. The question of the regulation of affects, the overflow or massive cleavage of which threatens the psyche with disorganization, even death, seems to be in the foreground.

### Emotional Regulation at the Neurobiological Level

The neurobiological bases of brain pleasure systems remain complex. Affective neuroscience has delineated three psychological components of reward: “liking” (hedonic impact), “wanting” (incentive salience), and learning (predictive associations and cognitions) (9), which are also dissociable at neurobiological level. These three processes can occur together at any time during the reward behavior cycle, although motivation tends to dominate the initial appetitive phase, while the hedonic impact dominates the next consumption phase, which can lead to satiety. Learning occurs throughout the cycle as it includes associations, representations and predictions of future rewards based on past experiences. Motivation seems to be supported by a circuit involving the ventral tegmental area (VTA), the nucleus accumbens, the striatum, the ventral pallidum, the amygdala, the lateral hypothalamus, the parabrachial nucleus and the prefrontal cortex; whereas hedonia only involves the nucleus accumbens shell, the ventral pallidum and the parabrachial nucleus. Thus, the dopaminergic system of VTA is involved in the motivation process but not in hedonic reactions (52). On the other hand, the endogenous opioid system of nucleus accumbens and ventral pallidum is mobilized by hedonic reactions.

The drugs all produce an increase in dopamine release in the nucleus accumbens (53), which increases the desire to consume the drug. Moreover, what is supposed to provide pleasure is the stimulation of opioid receptors, either directly (heroin) or indirectly through the release of endogenous opioids [psychostimulants (54), cannabis, alcohol, nicotine (55)].

The phasic activity of dopaminergic neurons in the VTA generates a learning signal when an unexpected reward occurs (56), so a drug diverts this system by generating a learning signal that ultimately drives to compulsive drug use. Repeated drug use leads to sensitization of the dopaminergic pathway (57) associated with the growing desire to consume; however, chronic drug use produces a tolerance of the opioid system, that is, it responds less and decreases its hedonic capacity (52). Thus, as drug use continues, its effects are reduced, resulting in increased frequency and doses, and ultimately, the onset of withdrawal symptoms. The repetition of drug use is largely related to the fact that the person needs to control their mood, to improve it, that is, to cope with his emotional vulnerability.

This vulnerability factor related to the subject has been demonstrated in rat experiments. Two subgroups were distinguished in the same batch of rats: those responding to stress by greater locomotor activity (HR: High Responder) and those responding by lower activity (LR: Low Responder). HRs consumed larger amounts of drugs, had higher dopaminergic activity, and secreted more corticosterone in response to stress (58). In addition, the stimulation of glucocorticoid receptors on dopaminoceptive neurons in the nucleus accumbens contributes to increased motivation for cocaine, probably by increasing dopamine release (59). The neurons of the nucleus accumbens exert, indeed, a control on VTA dopaminergic neurons. An increase in glucocorticoid receptor activity by stress, present or past, appears to be one of the conditions for the expression of increased vulnerability to drugs.

Persistence, even into adulthood, of the biological consequences related by trauma occurring in the first days of life, seems to be responsible for increased vulnerability to drugs. For example, in rats, the separation of neonates from the mother for 3 h per day during the first 14 days of life leads to an activation of histone deacetylases (HDAC), decrease in histone acetylation and an increase in morphine consumption in adulthood age. Treatment for 3 weeks with the HDAC inhibitor sodium valproate abolished HDAC activation together with the decrease in the acetylation levels of histone, and was accompanied with normalized morphine consumption (60). These data suggest that epigenetic regulations, which persist for a good part of life, would underlie the trace of early traumatic events.

In summary, drug consumption, initiated at the beginning to obtain pleasure, turns, after more or less numerous uses according to the vulnerability of the individual, into a process tending toward the habit of resorting to the drug each time an unpleasant affect occurs, ranging from boredom to deepest distress.

## Hypothetical Model of Drug Addiction

If we go back to the historicity of the addicts we met, there is first the trauma that occurs either very early, or even in the prehistory of the subject. One patient is subject to the narcissistic learning of a mother dependent on her own mother, while another inherits all the anguish of her maternal and paternal lineages abandoned to poverty. An addict is dealing with an unpredictable mother, while another is facing depressions over several generations. Finally, one is caught in a perverse bond to his parents, while another, unwanted, is abandoned. All these traumas refer to the narcissistically alienating effects of the object's influence, to an extremely fragile narcissistic configuration, which could be underpinned by epigenetic modifications of certain genes that induce hypersensitivity to stress.

Thus, a vulnerable background favoring the development of drug addiction would be that of a person forced to think prematurely in response to an environment poorly adjusted to the needs of the ego of an immature child. Thinking to adapt to this environment, and thus build a false self, which requires considerable psychic energy. Under these conditions, drug use is a means to return to the primary sensoriality, which is the primary True Self, heroin directly activating the receptors responsible for the feeling of well-being. The addiction would thus be set up as a counter-defensive process in a subject who has been subjected to inappropriate experiences of being in the world. Counter-defensive, because it breaks down the defenses put in place during the construction of the false self.

Conversely, in the course of a development that took place in a “good enough” environment, experiences of satisfaction gave rise to the learning of “rewarding” situations likely to fuel narcissism, and therefore without any need to consume drug to return to primary narcissism. Satisfaction experiences, such as a positive social connection, which is accompanied by endomorphin secretion.

The addictive process would involve several steps before to reach the actual addiction state, which would proceed as follows:

In adolescence, or even a little later, the encounter with drugs will be an experience of discovery of well-being. The defense systems put in place to protect the accumulated narcissistic faults are mitigated by the drugs. Through the discovery of intense sensations, the vulnerable subject has the illusion of taking power and becoming master at home, in particular, to control his body and his emotions. It is a taming of affect by a biological bypass. From passive submission to the malaise that dwells him, he passes to the active recourse of solitary pleasure.

However, when the use is more chronic, the effect of the drug changes, becomes less and less hedonic, and is more oriented to a search for normality. It is the relaxation provided that is now expected, relaxation that allows to move away from trauma. But to relax, regular intake of product is required, whose frequency depends on the stress that assails the person.

In a third step, the disappearance of the pleasurable subjective effects specific to drugs reveals a subjective effect of the consumption behavior itself, which would be close to pictograms (61), formal signifiers (62), but with the absence of response from the object. This prevents the primary symbolization from occurring and transforms the process into self-calming conducts or obsessive-compulsive behavior. The progressive automation of the motor sequence leading to consumption gives it, despite everything, a defensive function protecting the ego from the influx of internal or external excitations.

When the search for heroin or a substitute takes a lot of time and energy, then comes the solution of resorting to substitution treatment. However, treatment may be just a substitute for heroin deficiency, for example when there is no money to buy it. It can also be a transformation of the treatment into a means allowing continuing its drug addiction, by splitting the substitution into several doses taken daily and by administering it by the route previously used for heroin (injection or sniffing). In this case, the relaxation obtained is related only to the gesture of consumption, that is to say that there was an associative learning between the taking a product and the relaxation caused. This is similar to the placebo effect observed after waiting for a beneficial result after treatment. A hypoalgesic placebo, for example, releases endogenous opioids that act on the anterior, orbitofrontal, lateral prefrontal, and insular cingulate cortex, nucleus accumbens, amygdala, and periaqueductal gray matter (63). One of the mechanisms underlying this effect is conditioning learning (64). In addition, a placebo-reward hypothesis has been proposed, since many structures of the reward system are involved in the placebo effect. This hypothesis proposes that waiting for a beneficial treatment is considered as a reward (65). Thus, a substitute product consumed as a drug would be a placebo of the expected relaxation effect. For example, a patient who injected the opioid ligand buprenorphine three times a day survived the emptiness feeling that inhabit him the rest of his time. In the same way, another patient, sniffing part of his buprenorphine after his work day, got rid of the tiredness that overcame him when he returned home.

At the biological level, drug use activates the hedonic system (opioid of the nucleus accumbens and ventral pallidum) and the motivational system (mesocorticolimbic dopamine); these systems are recruited during all pleasurable activities. It can be assumed that the individual having access to other pleasant activities, will not systematically consume drugs to obtain this hedonism, this access to the true self. On the other hand, a vulnerability linked to early trauma can trigger addictive behavior, because of the greater sensitivity of glucocorticoid receptors triggered by stress. Once the consumption is installed, the modulations of the biological systems are multiple.

At first, it is a phenomenon of biological tolerance that is developed in the patient. Chronic administration of heroin or cocaine decreases the function of second messengers of opioid mu and delta receptors and dopamine D2 receptors. This disruption of the intracellular signal of the G_i/0_ inhibitory protein leads to an increase in the formation of cyclic adenosine monophosphate and protein kinase A in the nucleus accumbens. This sequence constitutes a critical neuroadaptation which results in an increase in drug use (66). This produces a tolerance which encourages to increase the doses and frequencies of consumption in order to obtain the same effect and to avoid withdrawal symptoms.

During a second phase, the consumer behavior, initially goal-directed, increasingly become elicited as stimulus-response habits by drug-associated conditioned stimuli. This transition from use to addiction depend upon shifts from ventral to dorsal striatal control over behavior, mediated in part by a serial connectivity between the striatum and midbrain dopamine systems, and upon the loss of prefrontal cortical inhibitory control over drug seeking habits (67). This transformation of the intentional act into habit brings into play neuronal plasticity, which is a normal physiological mechanism underlying, among other things, motor learning, but which could constitute also the first step toward pathological compulsion. Moreover, chronic use of addictive drugs elicits a persistent striatal hyperactivity, associated with an impairment in hippocampus-dependent forms of memory, which may lead to cognitive rigidity and a higher risk of relapse. Therefore, this mechanism could promote conditioning to the detriment of more flexible forms of memory (68–70). Finally, emotional and stressful events are potent modulators of striatum–hippocampus interactions: they promote habitual over cognitive forms of learning, through mineralocorticoids and adrenaline interaction (71). Of note, the amygdala plays a key role in orchestrating the switch from hippocampal to striatal learning (72, 73). Conversely, the synergistic actions of glucocorticoids and emotional arousal-induced noradrenergic activation is required for memory consolidation (74).

Although, the biological understanding of the mechanisms of drug addiction is still sparse, it opens up interesting avenues about the long-term effects of drugs of abuse. For example, does the mechanism of opiate receptor tolerance as expressed in the nucleus accumbens and possibly involved in hedonism, accompanied by the maintenance or sensitization of opiate receptors located in other areas of the brain and responsible for the feeling of relaxation procured by heroin? Alternatively, could this feeling of relaxation be produced by conditioned learning?

## Psychotherapeutic Perspectives

Drug addicts come to the CSPCA when the defensive solution of addiction fails, when they spend more time than they want to seek their product, especially when they are socially active, when their financial debt accumulates, when anguish invades them and when they feel that they have lost the control of their life. They come to consult when they experience secondary suffering caused by their addiction, while the primary suffering at the origin of their drug use is rarely considered. But their request concerns above all a treatment of substitution, and sometimes only that. Some patients trivialize their behavior, arguing “this is to do like the others.” Also, when the secondary suffering is treated by the substitution, reappears then the primary suffering that is likely to be a cause of relapse, of a new recourse to the self-medication by the drugs. Therefore, the use of drugs must be understood as a defense mechanism, a strategy of unconscious emotional regulation, for which function is to avoid, minimize or convert affects that are too difficult to tolerate (75).

Substitution treatment is often necessary to start health care. It allows social reintegration and medical follow-up. However, the combination of behavioral interventions with maintenance treatment is controversial (76). Half of the authors reported no benefit from adding counseling, cognitive-behavioral therapy or contingency management to buprenorphine, while the other half demonstrated the efficacy of behavioral interventions, particularly contingency management. This could be due to the substantial differences that exist among therapists in drug addict outcomes because the relational factors such as therapist empathy and therapeutic alliance can be significant determinants of addiction treatment outcome (77). To promote these relational factors, clinicians must begin by “taming” their addicted patient. For example, following an absence or a delay in an appointment, it is necessary to play on the resumption of appointment, because these defense mechanisms must be considered as part of addictive pathology. To register clinician in the reality, they attempt to “destroy” him and test his ability to survive (78) at their game of hide and seek. They can then settle in a space/time that they enjoy at their convenience, a space of “subjective security.” If this period of “taming” works, the regularity of the appointments is established, because the clinical encounter has turned into an “attractor”.

The first task to perform is a restoration of narcissism through a narcissistic accompaniment mode (49). They present themselves with all their inadequacies put forward, they fail to be on time at their appointment, and they cannot stop consuming. This negativity invades the space/time of the first interviews, the narcissistic suffering is present. They condemn themselves with much guilt that hides a primary shame. A “theory of evil” is advanced for which they would be solely responsible. It is important to tell them that addiction is not a vice but a pathology linked to their subjective history and to look for moments of resourcefulness with them. This step can take several months, or even years, but mixes with another work, that of reconstructing their history with their traumatic parts. Reflecting their emotion or naming their affects not expressed in their story is also a way to promote their symbolization. Indeed, in some cases, the hypothesis of the absence of a social biofeedback of the reflection of the affect (79) during infancy can be posed. For some, everything went well in childhood, which reinforces their idea that they have fallen into a deviant behavior; for others, the memories are nonexistent, their remembrance does not go back beyond the escapades of adolescence. Thus, the return to their infantile period makes them weave links that they had not imagined before.

The last work consists in working out the early traumas, mourning an ideal primary relationship, inserting prehistory into the narrative of its history in order to understand where the insufficiency of its primary objects came from.

To summarize, follow-up of addicted patients requires the interpretation their acting out as much as listening to what they say. It can only be done in a flexible setting accompanying relapses, a “malleable medium,” “made-to-measure” setting. One of therapy goals should therefore be to help dependent patients gradually build their adequate personal environment as a substitute for the drug, with a deep respect for where they are in their life course.

## Conclusion

By skimming through the genesis and functions of heroin addiction, several psychic processes underlying this psychopathology have been linked to biological mechanisms, although many “bridges” are still missing. In addition, can the hypotheses put forward extend to addictions toward other chemical or behavioral agents? Additional important research will be required to answer this question.

Our reflection on the genesis of the addiction made us identify the narcissistic and biological vulnerabilities that take root in the intra-uterine life. Then, early care experiences and emotional interactions are increasingly understood to have direct repercussions on brain organization, with the recognition that depending on the type of attachment developed, they will lead to resilience or deterioration of mental health.

Heroin activates the hedonic system, thus providing defensive function against trauma encountered throughout life. However, the opioid system is widely distributed throughout the brain, and the effects of exogenous opiates on opioid receptors (μ, δ, and κ) located in various brain regions requires further investigation. Opiates also have less pleasing effects such as nausea, dysphoria, or aversive hallucinations likely due to their action on kappa receptors. Understanding which effects depend on individual variability in kinetics and metabolism, and which ones are context-dependent remains a crucial challenge for opioid neuropharmacology.

Beyond a rough outline of the neurobiological bases of the psychopathological processes of drug addiction, the current article proposes a new approach of the drug addiction by highlighting the recourse to the recovery of a process of primary narcissism essential to life. This is made possible by the proximity of biological life and psychic life during an early developmental phase that causes the psychic processes to take root in the body. Finally, the present view of addiction calls for a therapeutic intervention that would be closer to the subject's experience, and the necessary patience to support and to take care of individuals suffering from opiate addiction.

## Author Contributions

HS contributes to the conception of the work, acquires, and interprets the data and drafts the work.

## Conflict of Interest

The author declares that the research was conducted in the absence of any commercial or financial relationships that could be construed as a potential conflict of interest.
